# Approaches for simplified HCV diagnostic algorithms

**DOI:** 10.1002/jia2.25058

**Published:** 2018-04-10

**Authors:** Slim Fourati, Jordan J Feld, Stéphane Chevaliez, Niklas Luhmann

**Affiliations:** ^1^ Department of Virology Henri Mondor Hospital National Reference Center for Viral Hepatitis B, C and delta D INSERMU955 Créteil France; ^2^ Toronto Centre for Liver Disease Sandra Rotman Centre for Global Health University of Toronto Toronto Canada; ^3^ Médecins du Monde France Paris France

**Keywords:** HCV, point‐of‐care, rapid diagnostic test, screening, diagnosis

## Abstract

**Introduction:**

In the light of the advances in HCV antiviral therapy, global control of HCV infection becomes feasible but depends on the capacity of countries to identify infected people and to offer them treatment. To achieve the WHO goal which targets a diagnosis rate of 90% by 2030, simplification of screening and diagnosis will be crucial.

**Methods:**

Published literature, unpublished data and expert consensus were used to determine key parameters, including point‐of‐care, rapid diagnostic testing, screening, the use of HCV core Ag and dried blood spots; starting from 2008 until November 2017. In addition, a manual search was undertaken to detect relevant papers or websites related to specific data from countries which underwent or are planning a programme of HCV elimination.

**Results:**

Several strategies have been developed and evaluated these last years to simplify and facilitate access to screening and diagnosis, the development of reliable HCV core antigen tests and new nucleic acid amplification technologies for use in decentralized settings. In high prevalence settings, a one‐step screening and diagnosis strategy could simplify diagnostic algorithms provided the cost is reduced. Finally, genotyping may no longer be required in the context of availability of pangenotypic antiviral therapy.

**Conclusions:**

Despite relevant advances in HCV screening and diagnosis, the overall diagnosis package is still too expensive today and efforts must be made to allow generalized implementation of reliable tests in low and middle income countries. These efforts will be key factors to foster a real public health approach to HCV elimination.

## 1 Introduction

Globally, more than 350 000 HCV‐infected individuals die each year from HCV, predominantly as result of decompensation of liver cirrhosis or hepatocellular carcinoma 1. Because chronic hepatitis C is often asymptomatic until advanced liver disease develops in many countries, a majority of infected persons are unaware of their infection 2. Today, in contrast to HIV, a generalized systematic approach to HCV testing has not been developed and adopted worldwide. Only national initiatives have been introduced. For instance, the Centers for Disease Control and Prevention (CDC) in the United States initially recommended serology testing in individuals with an identifiable risk factor for HCV infection 3 and has recently extended the indication of anti‐HCV antibody screening to all individuals born between 1945 and 1965, the so‐called “baby‐boomers”^4^. The European Centre for Disease Prevention and Control (ECDC) plans to issue guidance for reducing the transmission of HCV among vulnerable groups, and especially people who inject drugs (PWID), migrants and prisoners 5. National screening policies are being implemented across Europe. In France, healthcare professionals were recommended to offer hepatitis C screening to specified at‐risk patient groups, including haemodialysis patients, patients with a history of blood transfusion before 1991, individuals who either injected or sniffed drugs, persons with a history of incarceration, healthcare professionals after occupational exposure to potentially infected blood, persons having unprotected sex with multiple partners, and persons living with an HCV‐positive individual 6. These measures led to the identification of over 70% of infected patients in the country. More recently, a report from the French Minister of Health recommended systematic screening of men aged 18 to 60 years and pregnant women at the first prenatal visit, in order to diagnose the remaining 30%, provide therapy and control HCV infection over the next 10 years 7. Finally, in its recent guidelines for low‐ to middle‐income countries, the World Health Organization (WHO) recommended that HCV and HBV serology testing would be offered to individuals who are part of a population with high prevalence or have a history of risk exposure or behaviour.

Overall, the diagnostic rates in the general population are still low, even in countries that adopted routine, population‐based HCV screening as in France 8, 9, 10, 11, 12 A recent study assessing the prevalence of HCV in the EU estimated the number of viremic HCV infections in 2015 to be 3 238 000 while only an estimated 1 180 000 (95% UI: 1 003 000 to 1 357 000) individuals having already been diagnosed (36.4%) 13. The diagnostic rates can be as low as 31% in Czech Republic, 33% in Portugal and 16% in Turkey 14 and much lower in low‐to‐middle income countries (LMICs) 15. In the US, the diagnosis rate is estimated around 50% 16.

Barriers to large‐scale screening and diagnosis in the past can be explained, at least in part, by the complexity of algorithms, the costs of the required tests, the absence of reliable alternative tests to classical serological and molecular assays (such as point of care tests), the complexity of on‐treatment monitoring with ribavirin and interferon, the cost of treatment, and the limited efficacy of previous treatment regimens. Nowadays, the advent of highly effective direct antiviral agents (DAAs) and the availability of generic versions of these agents have changed the paradigm for the management of HCV infection. With the excellent safety and tolerability and high cure rates of DAAs (>95%), the major remaining barrier to treatment is the under‐diagnosis of HCV and limited access to treatment in the diagnosed population. DAA prices are starting to fall due to innovative approaches, to negotiations with industry, as well as continued development of new agents bringing competition to the marketplace.

Current screening of HCV infection is based on anti‐HCV antibody detection using a third‐generation enzyme immunoassay (EIA). Third‐generation EIA use a multiantigen format including antigens from the core, NS3, NS4 and NS5 regions; these tests show excellent sensitivity and specificity (>99%) and are considered reference standards to detect anti‐HCV antibodies 17, 18. Antibody detection serves only as a screening tool because, it is unable to identify individuals who have an active infection from those who have a resolved infection and are no longer viremic. The anti‐HCV antibody detection should then be followed by HCV RNA determination using nucleic acid amplification technologies (NAAT) and completed by HCV genotyping that is required for treatment decision‐making 19, 20, 21. However, such a complex set of diagnostic test is not reachable to people who have limited access to the health system (high‐income countries – PWID, people in prisons and other closed settings, sex workers and migrants) or in countries where the health system has poor infrastructure for testing (LMICs). In recent years, new tools have been developed to simplify screening, diagnosis and on‐treatment monitoring of HCV. These tests include point‐of‐care (POC) tests [immunological POCT tests with the rapid diagnostic test (RDT) and the non‐ immunological POC tests based on nucleic acid detection and quantification] and serological assays that detect and quantify HCV core antigen (HCV cAg) as an alternative to HCV RNA detection and quantification 22, 23, 24. In addition, the use of dried blood spot (DBS) that allow collecting blood on filter paper is a promising intervention to promote uptake of hepatitis C testing and linkage to care.

The primary goals of simplifying HCV screening and management are to better identify infected individuals, increase rates of retention and linkage to care and treatment, reduce the costs of diagnosis for patients and the healthcare system with the ultimate goal of reducing viral transmission at a population level, and progression of liver disease and hepatitis‐related mortality at an individual level. Dynamic HCV transmission models demonstrate that screening and treatment of HCV‐infected individuals in high prevalence settings can reduce the incidence of HCV by reducing HCV transmission (termed ‘‘cure as prevention”) 25, 26, 27. The first global health sector strategy on viral hepatitis that was adopted by WHO in May 2016 calls for a 90% diagnosis rate for the year 2030 globally 28 a goal that is unlikely to be achieved unless the screening and diagnostic algorithms for HCV can be markedly simplified in the near future 29.

The aim of this review is to provide a comprehensive overview of new simplified approaches for screening, diagnosis and monitoring HCV infection within different country‐specific settings and to describe key promising tools in future diagnostics.

## 2 Methods

Published literature, unpublished data and expert consensus were used to determine key parameters consisting of the following terms: sample type (oral fluid, fingerstick, venous blood), point‐of‐care, rapid diagnostic testing, screening, the use of HCV cAg and dried blood spots. In addition, a manual search was undertaken to detect relevant papers or websites related to specific data from countries, which planned or are planning a programme for HCV elimination. The literature search was limited to English language, available from 2008 until November 2017.

The study population was analysed in each study (general population or high risk population). The high risk population groups include men who have sex with men, sex workers, PWID and prisoners. Studies that only reported sensitivity or specificity, or those that only used reference assays for positive samples were excluded from the review.

## 3 Part 1 – Simplified approaches for screening HCV

With the emergence of highly efficacious antiviral treatment, the possibility of curing the vast majority of patients is now a realistic goal 29. To achieve all required steps in the care cascade, there is a need to increase community awareness of HCV and to simplify strategies for HCV screening. Until recently, most screening for HCV has been done within medical settings and relied on rather centralized laboratory structures, which can facilitate the patients’ referral to care; but such screening is obviously not sufficient as evidenced by the low screening rates in most countries 15, even those with highly effective healthcare systems. A recent modelling study of HCV disease burden in the Europe 13 estimated that screening needs to expand from diagnosis of 88 800 new cases annually in 2015 in Europe to 180,000 by 2025 to achieve the WHO target.

Much of the challenge lies in the fact that many populations with a high prevalence of HCV, such as people who inject drugs (PWID), sex workers, migrants, those with mental health issues and incarcerated individuals often have reduced access to care in traditional medical settings for many reasons. As such, even if screening occurs, linkage to and retention in care is often limited, greatly reducing the overall impact of screening efforts. In LMICs, the situation is compounded by lack of access to high quality medical facilities, particularly for non‐acute medical issues. As such, decentralized rapid test‐based screening will be crucial to improve screening and linkage in both high and low/middle income countries.

### 3.1 Screening access in difficult‐to‐reach population

Facilitating access to screening can be achieved by improving serological tests in terms of rapidity and simplicity of performance such as using easy‐to‐access samples like fingerstick capillary whole blood or oral fluid. Simpler tests are more appealing to those being tested and potentially allow testing to be done by less skilled individuals, greatly increasing testing capacity both in numbers and the locations in which it can be performed. Rapid diagnostic tests (RDTs) have been already been developed for HCV antibody screening, with significant advantages over classical enzyme immunoassays (EIA).

Technically, classical enzyme immunoassays (EIA) require laboratory infrastructure and expertise in their operation. In contrast, RDTs do not require investment in laboratory equipment with minimal maintenance costs and reagents. Therefore, RDTs are suited for decentralized settings to reach individuals at highest risk for HCV who may remain outside the traditional medical system. To be acceptable for screening, RDTs need to meet the high standard of traditional testing tools in terms of analytical performance for accuracy, reproducibility, sensitivity and specificity and in terms of clinical performances. Clinical performances of individual tests are heterogeneous and vary widely 30, 31. Comparisons have shown significant variability depending on the manufacturer, the sample type (i.e. fingerstick whole blood, oral fluid etc.) and pre‐analytical conditions (preservation conditions 32). For example, the clinical sensitivity for the OraQuick^®^ HCV rapid antibody test (OraSure Technologies, PA) was excellent in finger stick whole blood (99.4%) as well as in oral fluid (97.6%) while the Labmen HCV test (Turklab, Izmir, Turkey) showed poor sensitivity in fingerstick whole blood (63.1%) 33. In addition, all tests showed better positive and negative predictive values in studies that were conducted in developed countries than in developing countries 34 partly explained by variation in disease prevalence of the targeted population. Therefore, caution must be paid regarding poor‐quality test kits and reagents and independent studies must be conducted for “real‐life conditions” to ensure that the individual RDT approaches work in the specific setting where they will be used. As with standard EIA tests, WHO has taken leadership to evaluate new RDTs and to date have pre‐qualified two RDTs that have shown excellent clinical sensitivity and specificity compared to standard EIA assays (i.e. OraQuick HCV Rapid Antibody Test Kit and SD Bioline HCV).

### 3.2 Screening access in low‐and middle income settings

In LMICs, the use of classical serological enzyme immunoassays (EIA) for screening may be limited for many reasons including poor laboratory infrastructure, insufficient staff and/or insufficiently trained staff, and poor‐quality management systems for relaying results, all of which hamper the access to accurate and timely screening. In these contexts, point‐of‐care RDTs appear promising 33. Initiatives should be taken to decentralize testing and to improve the retention of patients both before and after testing.

Some countries (e.g. Georgia, Egypt) have already engaged partners to develop an efficacious prevention and control plan, which has led to an improved access to diagnostics and treatment for HCV‐infected individuals with severe disease. In Georgia, an estimated 5.4% of the adult population (approximately 150,000 persons) has chronic HCV infection, and most of them were unaware of their infection in 2015 (Georgia Ministry of Labour, Health, and Social Affairs, 2016) 35. Georgia initiated on the world's first programme to eliminate HCV, defined as a 90% reduction in HCV prevalence by 2020 36, 37. Control plans include ongoing HCV screening programs since 2015 provided at various settings at no cost (e.g. blood donors, pregnant women, hospitalized patient, PWID…). Persons who screen positive for HCV antibody are referred to the treatment programme for confirmation of chronic HCV; however, unlike initial screening, HCV RNA measurement is not free of charge. Offering free HCV confirmatory testing, support campaigns to expand public awareness will be crucial to achieve the goal of HCV elimination in the country. Information systems capable of linking screening and treatment data are being developed to improve efficiencies.

In Egypt, where the prevalence of hepatitis C virus (HCV) infection is the highest in the world 38, the immediate plans are to focus on treating HCV patients with liver cirrhosis identified in the past few years, followed by screening and treatment programs for high at‐risk population. The last stage will include national screening and treatment of patients from the general population.

As each region/country needs to plan its own approach by implementing adequate HCV testing, the experience from those countries who have taken the first step towards eliminating HCV can help pave the way for other countries experiencing high rates of HCV infection to undertake similar initiatives, and help curb the global epidemic of viral hepatitis.

### 3.3 Use of dried blood spot

Dried blood spot sampling is an interesting alternative collection method to collect whole blood specimens (either by capillary fingerstick or by venipuncture). The sample is easily transferred onto filter paper and can then be easily transported to a centralized laboratory where testing can take place. The two main advantages of this sample type which can facilitate the expanded access to screening and diagnosis is (i) the option to use fingerstick whole blood and (ii) the stability of the specimen allowing for simple transport (even by regular mail) without the need for an intact cold chain. Once in the central laboratory, DBS samples can be stored long‐term at −20° or −80°C. Once ready for testing, the sample is eluted off the filter paper using an appropriate buffer and the eluate can then be used in the same testing systems used for serum or plasma. One other major advantage of DBS is that multiple spots can be taken at once allowing for reflex HCV RNA testing on a second (or third) spot if the initial spot is positive for HCV antibodies thus avoiding the need to bring individuals back for confirmatory HCV RNA testing, which has consistently been shown to be a major drop‐off in the cascade of care of most settings. DBS is particularly attractive for difficult‐to‐reach populations. Because phlebotomy is not required, testing can be done by peer workers with limited training and venous access, often a challenge for PWID, is less of a concern. Recent studies have confirmed the improved acceptability of DBS over standard testing approaches 39. The actual testing from DBS samples uses traditional EIA approaches and as such a centralized experienced laboratory is required to process the specimen, however the ease of transport limits the need for multiple central laboratories.

The performance for detection of anti‐HCV antibodies in DBS specimens compared to plasma or serum specimens has shown excellent results with sensitivity and specificity generally over 95% 40. HCV RNA detection is somewhat less reliable, particularly at low viral titres because of the lower volume of sample acquired after elution off the DBS. A recent study 40 estimated that HCV RNA levels in whole‐blood specimens from DBS were lower by >1.5 log IU/ml on average than those in serum specimens; however, HCV RNA can be detected in almost all DBS. Depending on the NAAT used, the limit of detection of HCV RNA from DBS has been estimated between 58 IU/ml to more than 250 IU/ml 41, 42. While the absolute amount of HCV RNA should not be considered when quantification is performed on DBS, the qualitative HCV RNA results from DBS is reliable as long as HCV RNA levels are high as it is the case in >95% of patients at diagnosis and at relapse.

The main disadvantage of DBS is that the existing commercial assays have not been validated so far or received regulatory approval with this method of sample collection and transport. Some studies published detailed protocols on how to collect and analyze DBS 43. However, manufacturers have not yet provided specific instructions on how to use their assays with DBS (including processing methods, pre analytical treatment and cutoffs of interpretation) even if no claim for regulatory approval is made until today, which make quality control challenging. There is an urgent need for development of standardized protocols by manufacturers, as well as the elaboration of large studies on use of DBS conducted in different settings and with varying storage conditions (including areas with extreme wet conditions for RNA assessments).

### 3.4 Rapid multianalyte testing for other pathogens (HIV, HBV and syphilis)

Multianalyte testing refers to testing in the same specimen the detection of HCV along with other pathogens, for example, HIV, syphilis and/or HBV. Such an approach has several obvious advantages including the requirement for a lower specimen volume, fewer fingersticks if capillary whole blood is used, less time required than a series of tests and potentially other cost efficiencies. This approach is promising, but data on clinical sensitivity and specificity of the assays are still required as well as the evaluation of their impact on patient management 44, 45. The multianalyte approach has been touted primarily for screening of acute illnesses to help in outbreak settings but may have advantages for the screening of chronic diseases as well.

## 4 Part 2 – Simplified approaches for diagnosis

The complexity of current algorithms for HCV diagnosis limits the ability to advance large‐scale screening programmes. Even if screening can be done, limited capacity to specialty care is often an additional barrier to care. Simplifying strategies of diagnosis/management of HCV infection will enable the potential transfer of treatment care outside of classical specialty care (i.e. infectious diseases and hepatology clinics) for most patients, likely all but those with advanced liver disease. In addition, in LMICs, lack of simple and affordable HCV diagnostic solutions represents a restraint to ensure broad access to care, as current diagnostic tools are insufficiently developed. Innovations in technologies for point‐of‐care testing (using nanotechnologies, microfluidics, biosensors and/or synthetic biology) have led to the creation of chip‐sized laboratory systems that could be helpful in the future 46. While there has been huge focus on the cost of antiviral drugs, very little focus has been put on the cost of diagnostics. It is important to note that in many countries (e.g. Kenya), diagnostic tools (including monitoring and testing for cure) might be even more expensive than IFN‐free antiviral therapy. In Kenya, the estimated cost of a 3‐month treatment regimen with the generic drug of sofosbuvir will be around US$ 260 or less 47 while no efforts are made on reducing NAAT costs.

### 4.1 Tools for simplified diagnosis strategies

Probably the three main tools that should bring simplification to diagnosis of HCV are (i) the availability of reliable HCV cAg tests, (ii) the availability of new POC tests based on nucleic acid detection and quantification for use in decentralized settings; both of these assays could be used as one‐step procedure in diagnosing chronic HCV infection (in high prevalent population if the tests become less expensive) and (iii) the potential to skip the determination of the viral genotype in the context of availability of pangenotypic all oral DAA therapy.

#### 4.1.1 HCV Core Antigen test for Point of care testing

The HCV core antigen test (HCV cAg) targets the HCV nucleocapsid peptides 22 (p22), released from infected cells into plasma. HCV cAg is detected early and during the natural course of HCV infection as a surrogate marker of viral replication (Table 1) 23, 48. Nowadays, several assays are commercially available for specific detection of HCV cAg 49. The evaluation of these assays compared to HCV RNA was recently presented in a meta‐analysis 49 with Abbott HCV cAg assay and the ORTHO ELISA‐Ag test showing the highest sensitivity (up to 93.4% and 93.2% respectively), with very high specificity (>98%). In the French ANRS 12336 study 50, the Abbott HCV cAg quantification displayed high performances also in HIV and HBV co‐infected patients from Cameroon. Quantitative data are also available with the Abbott HCV cAg assay 23 showing a close correlation between HCV cAg and HCV RNA at viral loads (VL) above 3000 IU/ml. The lower cutoff for the assay corresponds to about 500 to 3000 IU/ml according to the HCV genotype, which will cover over the vast majority (>95%) of chronic HCV infections 51. The main benefit of using HCV cAg over molecular methods is the cost of testing. In the United Kingdom 52, NAAT cost was evaluated at 108 USD versus 23.4 USD for HCV cAg including kit, staff, and laboratory extras. In an Egyptian study 53, HCV NAAT test cost per individual (including equipment and personnel expenses) was estimated at 141 USD versus 19.8 USD for HCV cAg. A new point of care HCV cAg Assay (Daktari Diagnostics) is undergoing clinical evaluations; the launch is expected in 2018. Daktari Diagnostics announces the test is likely to cost 10 to 20 USD 54.

**Table 1:** Summary of the main characteristics of virological tools used for simplified diagnosis strategies

	HCV RNA using NAAT	HCV RNA using DBS	HCV RNA POCT	HCV c Ag
Analytical performances	Excellent sensitivity <25 IU/ml	Should only be used as a qualitative result	Expected to be excellent. Need to be determined in real life settings	Equivalent to 500 to 3000 IU/ml, according to the HCV genotype
Target population	Centralized settings” High income countries	Lack of access to sites or nearby laboratory facilities for NAAT Persons with poor venous access (e.g. in drug treatment programs, prisons).	Lack of access to sites or nearby laboratory facilities for NAAT if using fingerstick: Persons with poor venous access (e.g. in drug treatment programs, prisons).	Centralized settings Low and middle income countries
Specimen type	Serum/plasma requires venipuncture to obtain specimen	Fingerstick capillary whole blood samples	Whole blood samples but more data are warranted	Serum requires venipuncture to obtain specimen. Whole blood from DBS but sensitivity is poor
Time of result	Time to result: several hours/days and generally batched as one run	Several days	<120 min	<60 min
Laboratory infrastructure	Requires trained laboratory technician Requires laboratory facilities and equipment	Can be performed in decentralized settings	Can be performed in decentralized settings	Requires laboratory facilities and equipment
Standardization		Need for development of standardized protocols by manufacturers		

NAAT, nucleic acid amplification technologies; POC, point‐of‐care; HCV cAg , HCV core antigen.

Other advantages of HCV cAg over NAAT are the following: serological marker stable at room temperature for 96 hours, short time to obtain (less than 60 minutes) 22. However, the use of HCV cAg in LMICs still faces the problem of the availability of quite sophisticated laboratory equipment; therefore, point‐of‐care HCV cAg, difficult to implement, are still under development.

In summary, HCV cAg represents a reliable HCV diagnosis tool and, being less costly than viral load tests, should facilitate HCV screening and access to care. However, this solution depends on the presence of centralized laboratory machines. In addition, caution must be paid to some limitations of the assay: most studies were conducted in high resource areas within reference laboratories where data for genotypes 4, 5 and 6 are missing 49. In addition, false negative results can be detected in genotype 3 infected patients, explained by viral polymorphisms failing to detect HCV cAg 55.

#### 4.1.2 New NAATs for Point of care diagnosis

Quantitative NAAT is widely used for measuring viral load, identifying those in need of treatment, and to assess treatment response. In the era of short‐course, highly effective therapy, there is less need for quantification of HCV RNA for HCV management. Therefore, qualitative assays, in theory less expensive, can replace quantitative NAAT, particularly in LMICs. In addition, new random access NAAT technologies offer potential for POC diagnosis 56, 57. A random access system eliminates the need for batch processing and automates all aspects of nucleic acid testing in a single step. The system integrates sample introduction, nucleic acid extraction, reaction setup, and real‐time PCR amplification for the quantitative determination of HCV RNA in human plasma/serum specimens. Such a process allows for a rapid response, which is ideal for a single visit diagnosis while using a technology that does not require specific laboratory infrastructure or expertise in its operation. The system is simple enough to be performed reliably by individuals without a background in nucleic‐acid diagnostics or virology. Such facilities should be ideal as a two‐step strategy after screening with RDTs in places where centralized laboratories are not available and could theoretically enable a one‐step diagnosis if price comes down. Until today, approved point‐of‐care HCV RNA assays require venipuncture 56, 57, which may be challenging in settings without access to phlebotomists or among people who inject drugs (PWID), due to poor venous access. The Xpert HCV Viral Load test (Cepheid), a WHO prequalified HCV NAAT test, has been recently shown to accurately detect active infection from fingerstick capillary whole blood samples 39. Although promising, it is still unclear whether a NAAT assay in a decentralized setting can achieve a price cheap enough to be used as a first‐line assay. Cost‐effectiveness studies are urgently needed to determine in which settings a one‐step versus two‐step, with RNA or HCV cAg quantification, laboratory or POC tests would be cost‐effective.

#### 4.1.3 Skipping HCV genotyping?

Once diagnosis is confirmed, the current algorithm of HCV management requires HCV genotyping before therapy is initiated. However, with the pangenotypic success achieved using next‐generation DAAs (e.g. sofosbuvir/velpatasvir, glecaprevir/pibrentasvir) with very high rates of SVR in different clinical conditions (naïve or pretreated patients, with or without liver cirrhosis), pre‐genotyping may no longer be required in the future. Whether genotyping can also be skipped when first‐generation DAAs (e.g. sofosbuvir/daclatasvir or generics) will be used in LMICs is questionable (at least in cirrhotic patients), as genotype 3 cirrhotic patients need longer duration of treatment and/or the addition or ribavirin when this combination is used 21.

### 4.2 Improving HCV testing by a one‐sample strategy

A significant proportion of anti‐HCV antibody‐positive patients fails to have a confirmatory test in difficult‐to‐reach populations and are lost to follow‐up 58, 59, 60, 61. To avoid this, one strategy consists of testing both anti‐HCV antibodies and a confirmatory test (for those with anti‐HCV reactive) from the same blood sampling performed on the same day, using a venous blood sample (which requires a centralized laboratory but is already a first step into simplification the cascade of care) or DBS sampling with multiple spots taken (see above “Use of dried blood spot (DBS) specimens for HCV screening”).

When DBS sampling is used, HCV RNA NAAT assays are preferred as confirmatory tests over HCV cAg; indeed HCV cAg testing has been shown to possibly have lower sensitivity on DBS compared to serum when only one spot is used 40.

The choice of whether to use DBS sampling for HCV serology or NAAT or both will depend on the healthcare setting and infrastructure, and epidemiological context. Different strategies can be suggested with varying combinations:

1. DBS EIA serology + DBS NAAT (context: No RDTs are available in decentralized settings; difficult‐to‐reach populations and difficulty in venipuncture);
2. RDT serology + DBS NAAT (context: RDTs are available in decentralized settings but no access to decentralized NAAT)
3. RDT serology + POCT NAAT (context: RDTs and non‐immunological POCTs are available in decentralized settings)
4. EIA serology + plasma/serum‐based NAAT (context: high income countries; large hospitals).

An interesting alternative would be a biphasic strategy with first testing anti‐HCV antibody (+) patients with HCV cAg, and reserving NAAT only for those who are anti‐HCV antibody (+) but HCV cAg (−). This strategy has been evaluated in a study performed in Toronto (Canada) to determine relapse in HCV infected patients treated with DAAs 62. In this context, the study showed that the use of HCV cAg could eliminate >75% of HCV RNA tests. A recent study 63 further indicates that this biphasic strategy is cost‐effective in the context of diagnosing HCV infection and is feasible in LMICs.

### 4.3 Improving HCV testing by a one‐step screening

**Figure 1:**
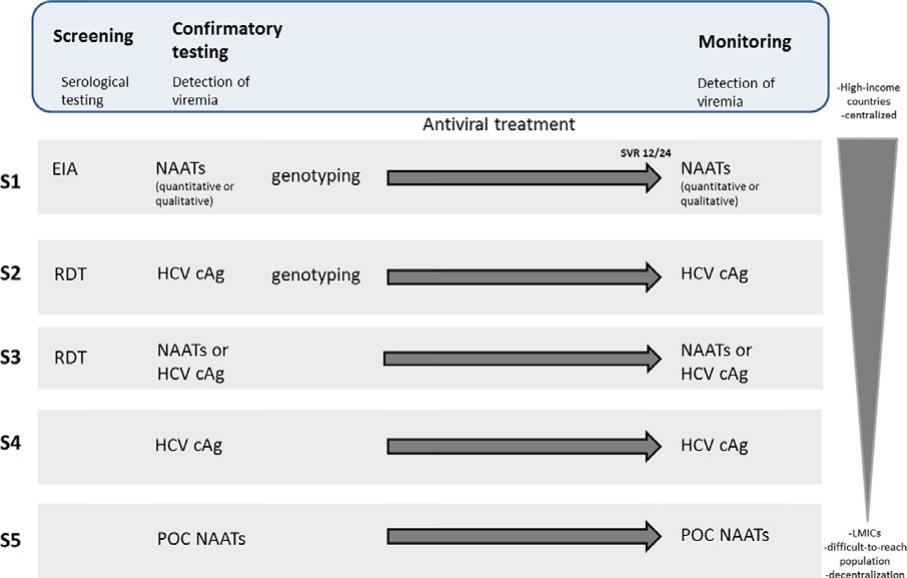
Proposed simplified algorithms using serological and virological tools for HCV screening, diagnosis and treatment follow‐up. Several strategies are suggested with varying combinations (S1 to S4). EIA, enzyme immunoassay; RDT, rapid diagnostic test; NAAT, nucleic acid amplification technologies; POC, point‐of‐care; HCV cAg, HCV core antigen.

To further simplify the diagnosis pathway, the ideal future algorithm would only require one test for both screening and diagnosing HCV. This test is preferably HCV cAg because of reduced costs or NAAT for HCV RNA (Figure 1) if these assays become affordable and in high prevalence settings, where the strategy should be cost effective. If such HCV cAg and/or NAAT assays are used at point‐of‐care settings, this should further improve access to early diagnosis and linkage to care for treatment services, in addition to reducing loss to follow‐up.

## 5 Part 3 – Simplified approaches for monitoring

The advent of interferon‐free DAA therapies has significantly simplified monitoring treatment. In addition to the fact that pangenotypic DAA combinations could eliminate the need for genotyping before starting treatment, there is no further need to monitor viral load during treatment 1 Using new DAAs, the levels of viral load decline no longer correlate with response and the number of virological tests can be reduced to a single post treatment virological test to assess cure (i.e. 12 or 24 weeks post treatment SVR). In this context, a second aspect of simplification is to perform the same test for diagnosis and for monitoring (i.e. either HCV cAg or NAAT for HCV RNA if affordable).

There remains some debate about the ability of HCV cAg to assess response to DAA treatment. There is emerging data supporting very good performances of the ARCHITECT assay as a test of cure 64. In a cohort of 181 patients (62% of whom achieved SVR) 62, HCV cAg was shown to determine SVR with very high accuracy when compared to HCV RNA in HCV‐infected patients receiving DAA treatment (concordance between Ag and HCV RNA for determination of SVR was estimated at 97.3%). Similarly, in a cohort of 411 HCV genotype‐1 patients with a pre‐treatment HCV RNA level >50,000 IU/ml who received a DAA‐containing regimen 24, concordance between HCV RNA levels and HCV cAg was 99.24% at follow‐up visit 12 weeks after the treatment cessation. Because HCV replication is likely to be higher in patients who relapse late after virological failure, HCV cAg testing might be even more sensitive in detecting treatment failures when performed at W24 post treatment compared to W12 post treatment. However, studies are needed to confirm this strategy. Specificity in anti‐HCV positive HCV RNA negative samples tested was 100%. Despite these encouraging results, the clinical performance of HCV cAg to confirm SVR at completion of therapy should be further investigated in particular in specific populations (e.g. HBV/HIV co‐infected patients, or infected with HCV genotypes 4, 5 and 6).

## 6 Conclusions

Control of HCV will strongly depend on the capacity of countries to identify people who live with chronic hepatitis C and to offer them treatment. Tools for simplified screening and on and post‐treatment monitoring will be critical to make such efforts feasible. Currently costs and complexity of diagnostics algorithm are notable and important barriers to screening and treatment monitoring. With the implementation of new treatment regimens, the current pathway for HCV diagnosis can conceivably be simplified from three tests (serology, NAAT and genotyping) to one or two tests if random‐access NAAT assays or reliable HCV cAg assays become affordable, particularly in high prevalence settings. A second aspect of simplification is to use the same test for diagnosis and monitoring (one single point at follow‐up visit 12 to 24 weeks). Finally, the capacity for decentralization including the use of new random‐access NAAT for HCV RNA will be crucial to further simplify diagnosis and monitoring. The future challenge will be to implement different diagnostic algorithms in different countries, based on the experience of similar countries who have already taken the first steps towards controlling HCV and explore innovative approaches to reduce the cost of these tests through large‐scale projects in different contexts (e.g. HIV or HBV co‐infection, difficult‐to‐reach population).

## Competing interests

SF: scientific consulting from Abbvie, Beckman. JF: grant funding from Abbvie, Abbott, Gilead, Janssen, Merck and Scientific Consulting from Abbvie, Contravir, Gilead and Merck. SC: Grant funding from Gilead, ANRS, Ministry of Health; Scientific Consulting from Abbvie, Gilead, Merck, Abbott Diagnostics, Hologic. NL: none to declare.

## Authors’ contributions:

SF and NL wrote the draft of the outline. SF wrote the first draft of the manuscript. JF, SC and NL critically reviewed the manuscript and provided input accordingly.

## Funding

None.
